# Plumbagin Exhibits Genotoxicity and Induces G2/M Cell Cycle Arrest via ROS-Mediated Oxidative Stress and Activation of ATM-p53 Signaling Pathway in Hepatocellular Cells

**DOI:** 10.3390/ijms24076279

**Published:** 2023-03-27

**Authors:** Huan Liu, Wenchao Zhang, Lijie Jin, Shasha Liu, Liying Liang, Yanfei Wei

**Affiliations:** 1 Laboratory of Medical Molecular Biology, The First Affiliated Hospital of Guangxi University of Chinese Medicine, Nanning 530024, China; liuchujie@yeah.net (H.L.);; 2 Guangxi Key Laboratory of Molecular Biology of Preventive Medicine of Traditional Chinese Medicine, Nanning 530024, China; 3 Research Center for Non-Food Biorefinery, Guangxi Academy of Science, Nanning 530001, China; 4 Department of Physiology, Guangxi University of Chinese Medicine, Nanning 530200, China

**Keywords:** plumbagin, cell cycle arrest, ROS, DNA damage, ATM/p53 pathway

## Abstract

Plumbagin (5-hydroxy-2-methyl-1,4-naphthoquinone, PLB), a naturally occurring naphthoquinone mainly isolated from the plant *Plumbago zeylanica* L., has been proven to possess anticancer activities towards multiple types of cancer. Although there has been an increasing amount of research regarding its anticancer effects, the association between oxidative stress, genotoxicity and the cell cycle arrest induced by PLB still remains unclear. Therefore, it is important to investigate their potential connections and the involvement of DNA damage and the ataxia telangiectasia mutated protein (ATM)-p53 signaling pathway in PLB’s anticancer mechanism. The present study showed that PLB exposure significantly reduced HCC cell viability and colony formation. In addition, PLB-induced G2/M cell cycle arrest, oxidative stress, and DNA damage was detected, which could be almost blocked by NAC pretreatment. PLB could trigger a DNA damage response by activating cell cycle checkpoints such as ATM, checkpoint kinase 1 (Chk1), checkpoint kinase 2 (Chk2) and p53. Meanwhile, the key modulator of the G2/M transition factor, Cell Division Cycle 25C (cdc25C), was significantly downregulated in an ROS-dependent manner. Furthermore, pretreatment with ATM and p53 inhibitors (KU55933 and Pifithrin-α) could reduce the occurrence of G2/M cell cycle arrest by inhibiting the activation of the ATM-p53 pathway. Taken together, these results indicate that ROS-mediated oxidative stress plays a key role in PLB-induced G2/M cell cycle arrest mediated by the ATM-p53 pathway.

## 1. Introduction

Hepatocellular carcinoma (HCC), along with other types of primary liver cancers, including intrahepatic cholangiocarcinoma (ICC) and combined hepatocellular cholangiocarcinoma (cHCC-CCA), ranks as the third leading cause of cancer-related death worldwide [1]. HCC accounts for 75–85% of the primary liver cancer cases, and its high recurrence and metastatic characteristics largely restrict the prognosis and long-term survival of patients [1,2]. In recent years, traditional Chinese herbal medicine and its bioactive components have emerged as a noticeable choice due to its multilevel, multitarget and synergistic effects in HCC treatment [3,4,5]. Therefore, there is a critical need to explore and evaluate possible alternative anticancer strategies, especially for advanced HCC treatment.

Plumbagin (PLB) is a natural naphthoquinone compound, which mainly originates from the root of *Plumbago zeylanica* L. This plant’s medicinal properties have been well documented in Chinese and other Asian countries, where it has been used in traditional medicine [6]. In China, this plant has long been used by the Uyghur and Yao nationalities in the treatment of diseases such as rheumatic pain, acariasis and even snake bites. In recent decades, plumbagin has attracted much attention due to its anticancer, antiviral and antimicrobial activities [7,8,9]. Regarding its anticancer properties, many studies have demonstrated that plumbagin exhibits significantly inhibitory effects on lung cancer, breast cancer, prostate cancer and many other kinds of malignant tumors [8,10,11,12]. Meanwhile, many mechanism studies have ascribed its anticancer activities to its ability to induce apoptosis and autophagy, the disruption of cell cycle regulation and the inhibition of invasion or metastasis [13]. In HCC, previous studies have shown that plumbagin could induce autophagy and apoptosis in HCC cells in vitro and in vivo, and its proapoptotic ability was found to be related to caspase 3/vimentin-mediated epithelial–mesenchymal transition (EMT) [14,15]. Recently, a network-pharmacology-based study delineated the importance of reactive oxygen species (ROS) production and the accompanying oxidative stress regarding PLB’s anti-HCC effects [16]. Nevertheless, a comprehensive anti-HCC pharmacological mechanism, especially the fundamental role played by the ROS-induced oxidative stress triggered by PLB, remains unclear.

Cancer is considered to be a group of diseases in which cells proliferate continuously and excessively. To satisfy this uncontrolled proliferation, the ROS level in cancer cells is usually much higher than that in normal cells, and serves as a signal activator for different pathways that are essential for cancer transformation and tumorigenesis [17]. However, ROS are also a well-known source of DNA damage and affect the DNA damage response (DDR) [18]. Activation of this response involves signaling to cell cycle arrest to allow for repair and to prevent DNA damage from being copied (G1 and S-phase checkpoint) or transmitted to the next generation (G2/M checkpoint) [19]. If the ROS-triggered DNA damage is irreparable, it is more likely to result in cellular programmed cell death in cancer cells than in normal cells [20]. Thus, combining traditional treatments with ROS-inducing agents is considered a promising strategy in cancer therapy [21]. Plumbagin is considered to be a robust ROS inducer, and excessive ROS production is often observed in cancer cells that are exposed to PLB [8,13]. In addition, G2/M cell cycle arrest is observed in many studies, indicating DNA damage in cancer cells that occurs after PLB treatment [8]. Regarding HCC, G2/M cell cycle arrest and elevated ROS production have recently been reported, and were regarded as the consequence of DNA damage and the increased expression of thioredoxin reductase-1 (TrxR-1) and heme oxygenase (HO)-1 (HO-1) [22,23]. However, no detailed correlation between ROS-mediated DNA damage and subsequent cell cycle arrest or cell death has been revealed to date.

This study aimed to evaluate the involvement of ROS-mediated oxidative stress and DNA damage response in PLB-triggered cell cycle arrest. For this purpose, the relationship between ROS-mediated oxidative stress, DNA damage, cell cycle arrest and signaling transduction of the DDR caused by plumbagin were investigated in HCC cells. In addition, the correlation between ROS-mediated oxidative stress and PLB’s cytotoxicity effects was also evaluated using antioxidant pretreatment. All these results will broaden our understanding of the key role that ROS-mediated oxidative stress plays in anti-HCC pharmacological mechanisms.

## 2. Results

### 2.1. Working Concentration Determination of PLB on HCC Cells

**Figure 1:**
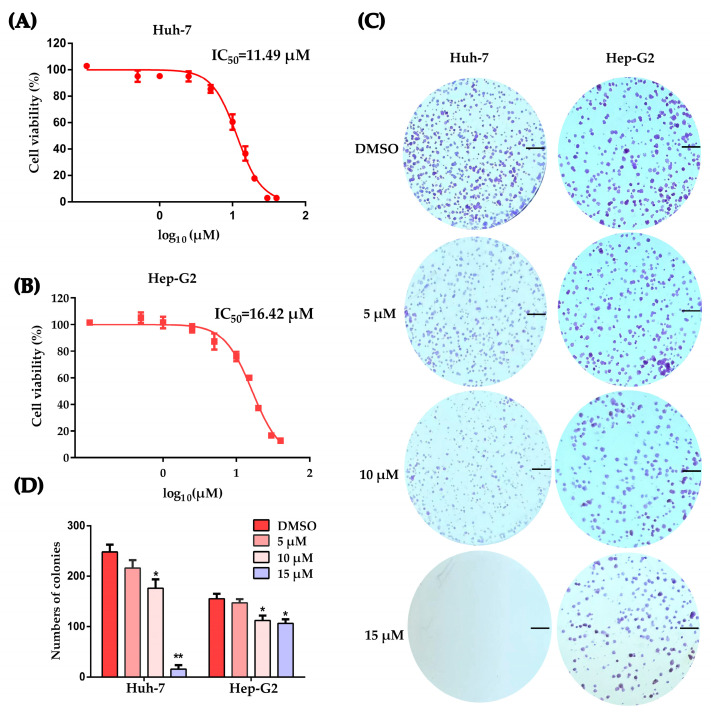
Determining the working concentration of plumbagin on HCC cells. (**A**,**B**) Huh-7 and Hep-−G2 cells were treated with plumbagin at concentrations of 0.1~40 μM for 12 h. Cell viability was tested by CCK-8 kit. Three replicates were set for this test and three independent experiments were performed. The data are shown as the mean ± SD. (**C**,**D**) Representative plates with colony formation assay, scale bar = 5 mm. Results are expressed as the numbers of counted colonies and compared to the DMSO group using *t*-test, * *p* < 0.05, ** *p* < 0.01 compared to the DMSO group.

It was previously demonstrated that plumbagin inhibits the proliferation of HCC cell lines such as SMMC-7721, BEL-7404 and LM3 [16,24]. To select the working concentrations, Huh-7 and Hep-G2 cells were treated by PLB at different concentrations for 12 h. Cell counting kit-8 (CCK-8) analysis results revealed that the concentration dependently inhibited the proliferation of HCC cells with IC_50_ values of 11.49 μM for Huh-7 cells and 16.42 μM for Hep-G2 cells (Figure 1A,B). To investigate the effects of PLB on colony formation, HCC cells were treated by PLB at the indicated concentrations for 12 h and subsequently cultured for 7 days. The results showed that HCC cells’ colony formation ability was also significantly suppressed (Figure 1C,D).

### 2.2. Plumbagin Induces G2/M Cell Cycle Arrest of HCC Cells

**Figure 2:**
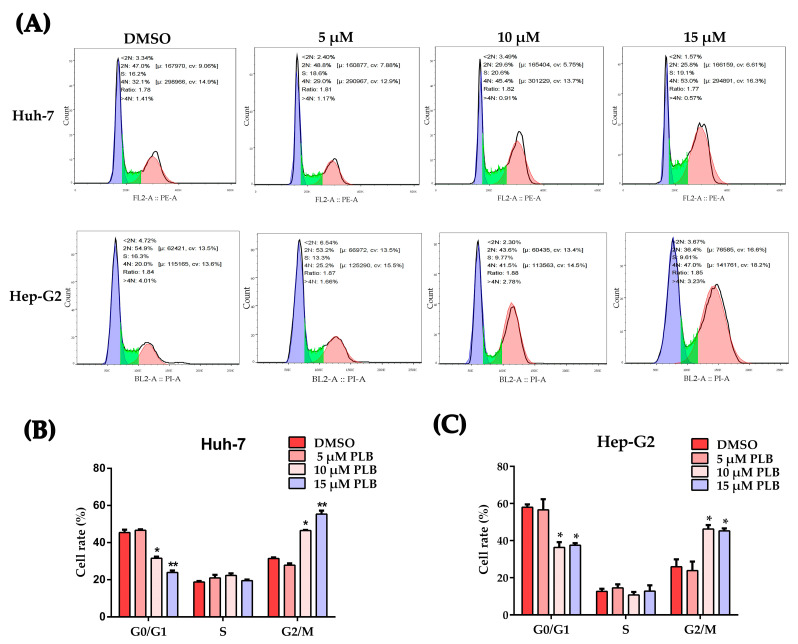
Plumbagin-triggered G2/M cell cycle arrest in HCC cells. (**A**) Representative images from the flow cytometry after treatment by plumbagin for 12 h. The colors purple, green and pink represent G0/G1, S and G2/M phase, respectively. (**B**,**C**) Percentage of the cells at different cell cycle stage was analyzed based on the flow cytometry results. * *p <* 0.05, ** *p <* 0.01 compared to the DMSO group; three independent experiments were performed.

Huh-7 and Hep-G2 cells were treated by plumbagin at the determined concentrations for 12 h and collected for analysis by flow cytometry. The results in Figure 2A demonstrated that plumbagin significantly induced G2/M cell cycle arrest. Meanwhile, cells at the G0/G1 stage were significantly decreased (Figure 2B,C).

### 2.3. Plumbagin Increased Oxidative Stress in HCC Cells

**Figure 3:**
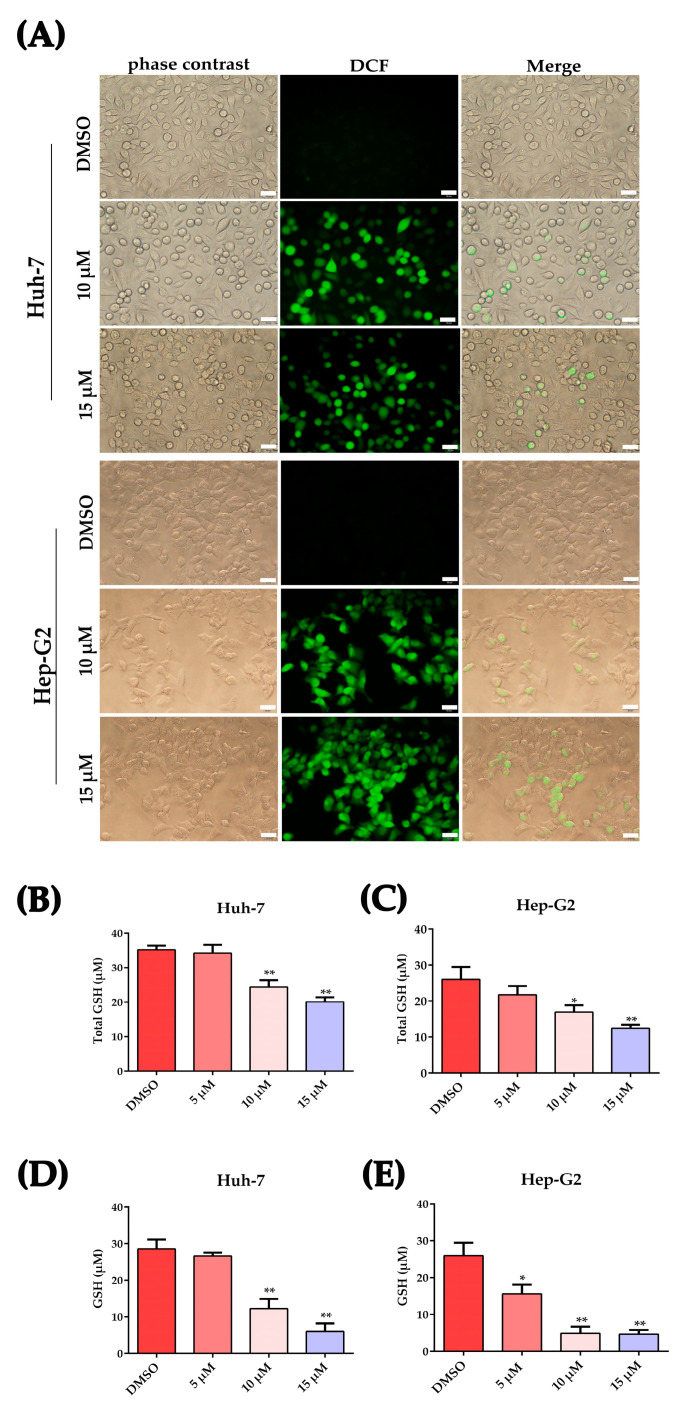
Plumbagin-induced oxidative stress in HCC cells. (**A**) Detection of ROS in HCC cells after plumbagin treatment. Nonblooming DCFH probes were oxidated by intracellular ROS and yielded a highly fluorescent product—DCF, which could be detected by fluorescent microscopy. ROS increased in Huh-7 or Hep-G2 cells after 12 h treatment with 10 μM and 15 μM plumbagin. Scale bar = 20 μm. (**B**,**C**) The total GSH level in HCC cells under PLB treatment. HCC cells were treated by different concentrations of PLB; then, the total GSH level was measured with the GSH and GSSG Assay kit (Beyotime, Shanghai, China). The data are expressed as the mean ± SD of three independent samples. * *p <* 0.05; ** *p <* 0.01 compared to the DMSO group. (**D**,**E**) The alterations in GSH relative content in cells after PLB treatment. After PLB treatment for 12 h, the cells were lysed and the GSSG level was tested using the kit mentioned above. GSH levels were further calculated following the instructions. The data are expressed as the mean ± SD of three independent samples. * *p <* 0.05, ** *p <* 0.01 compared to the DMSO group.

Oxidative stress refers to elevated intracellular levels of ROS, which are by-products of biological reactions caused by energy production. Here, we detected the cellular ROS and glutathione (GSH)/oxidized glutathione disulfide (GSSH) levels to determine if oxidative stress was induced by plumbagin in HCC cells. As shown in Figure 3A, HCC cells induced elevated ROS levels after 12 h treatment with 10 μM and 15 μM plumbagin. Glutathione peroxidase, which oxidizes the cysteine residues in GSH to disulfide bonds and yields GSSG, can be converted back to GSH under the direction of glutathione reductase with the aid of NADPH. As shown in Figure 3B–E, the total GSH content decreased in a concentration-dependent way after incubation with PLB for 12 h, the same trend as that shown for the reduced GSH content.

### 2.4. Plumbagin Induces DNA Damage in HCC Cells

**Figure 4:**
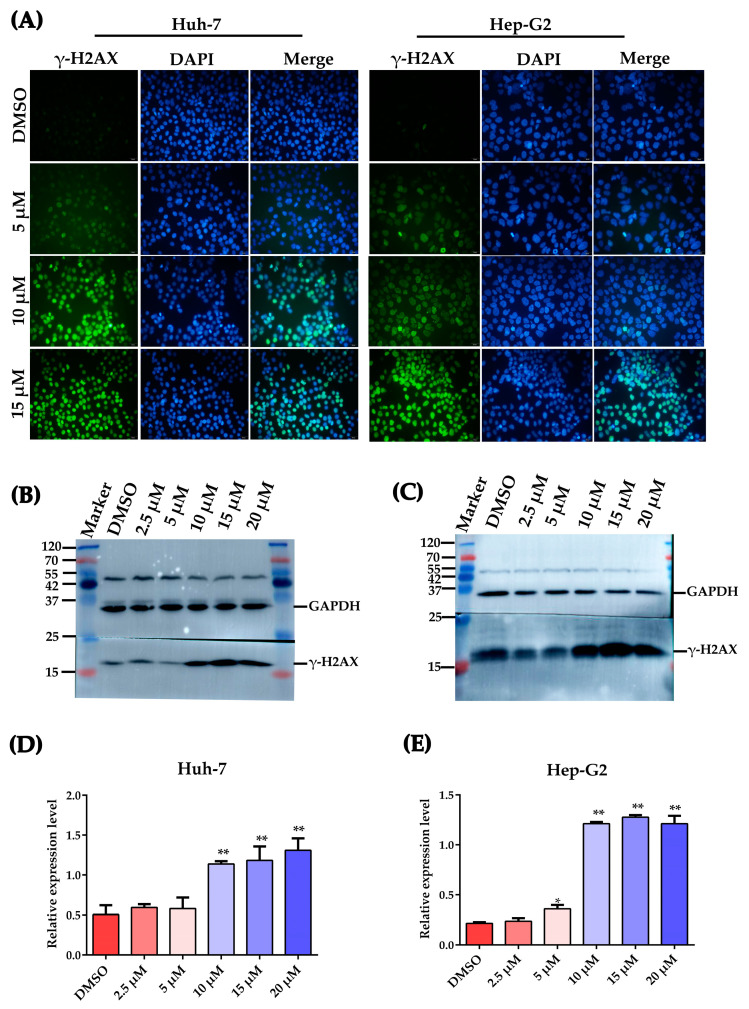
Plumbagin-induced DNA damage in HCC Cells. (**A**) Examples of pictures captured by fluorescent microscopy. The DNA damage marker γ-H2AX expression was revealed by green fluorescent signal, and the cellular nucleus is represented as blue fluorescence. Scale bar = 20 μm. (**B**,**C**) Western blot analysis of the expression levels of γ-H2AX after PLB treatment for 12 h. (**D**,**E**) Gray value statistical analysis of the relative expression levels of γ-H2AX after plumbagin treatment at different concentrations. * *p <* 0.05, ** *p <* 0.01 compared to the DMSO group; the data are expressed as the mean ± SD of three independent experiments.

Ser-139 residue phosphorylation of the histone variant H2AX, which forms γ-H2AX, is a sensitive molecular marker of DNA damage and repair [25,26]. γ-H2AX levels in Huh-7 and Hep-G2 cells were evaluated after plumbagin treatment for 12 h. As shown in Figure 4A, IFA results showed that plumbagin treatment could strongly activate the phosphorylation of H2AX at Ser-139; the cell rate containing γ-H2AX foci increased with the concentration of plumbagin treatment. Meanwhile, Western blot results also displayed increased γ-H2AX levels after PLB treatment. As shown in Figure 4B,C, the expression level of γ-H2AX significantly increased when the PLB level increased to 10 μM.

### 2.5. Plumbagin Activates DNA Damage Response in HCC Cells

**Figure 5:**
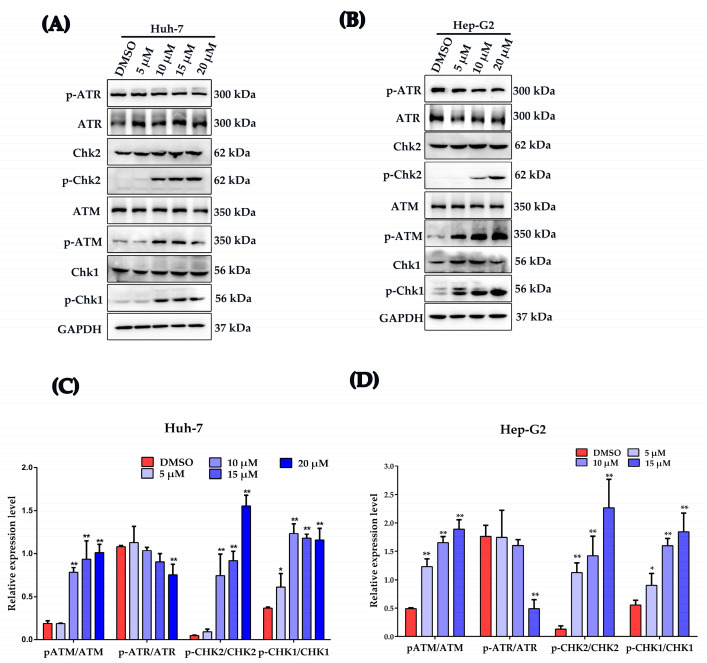
Plumbagin modulates the DNA damage response of HCC cells. (**A**,**B**) Huh-7 and Hep-G2 cells were dose-dependently treated by plumbagin and the expression of total ATM, p-ATM (Ser1981), total Chk2, p-Chk2 (Thr68), total ATR, p-ATR (Ser428), total Chk1 and p-Chk1 (Ser345) were analyzed by Western blot. (**C**,**D**) Gray value statistical analysis of the indicated proteins. * *p <* 0.05, ** *p <* 0.01 compared to the DMSO group; three independent experiments were performed.

In response to DNA damage, a network of events, including DNA damage recognition, activation of checkpoints and cell cycle arrest, were activated [19,27]. Western blot was used to investigate the influence of plumbagin on the activation of the main DNA damage checkpoints. As shown in Figure 5A–D, the key regulators of DDR sensor Ataxia telangiectasia mutated protein (ATM), checkpoint kinase 2 (Chk2) and checkpoint kinase 1 (Chk1) were significantly activated by plumbagin; however, another DDR sensor Ataxia telangiectasia mutated and Rad3-related protein (ATR) was downregulated by PLB at 15 μM.

### 2.6. NAC Treatment Inhibits the Cytotoxicity and DNA Damage Induced by Plumbagin

**Figure 6:**
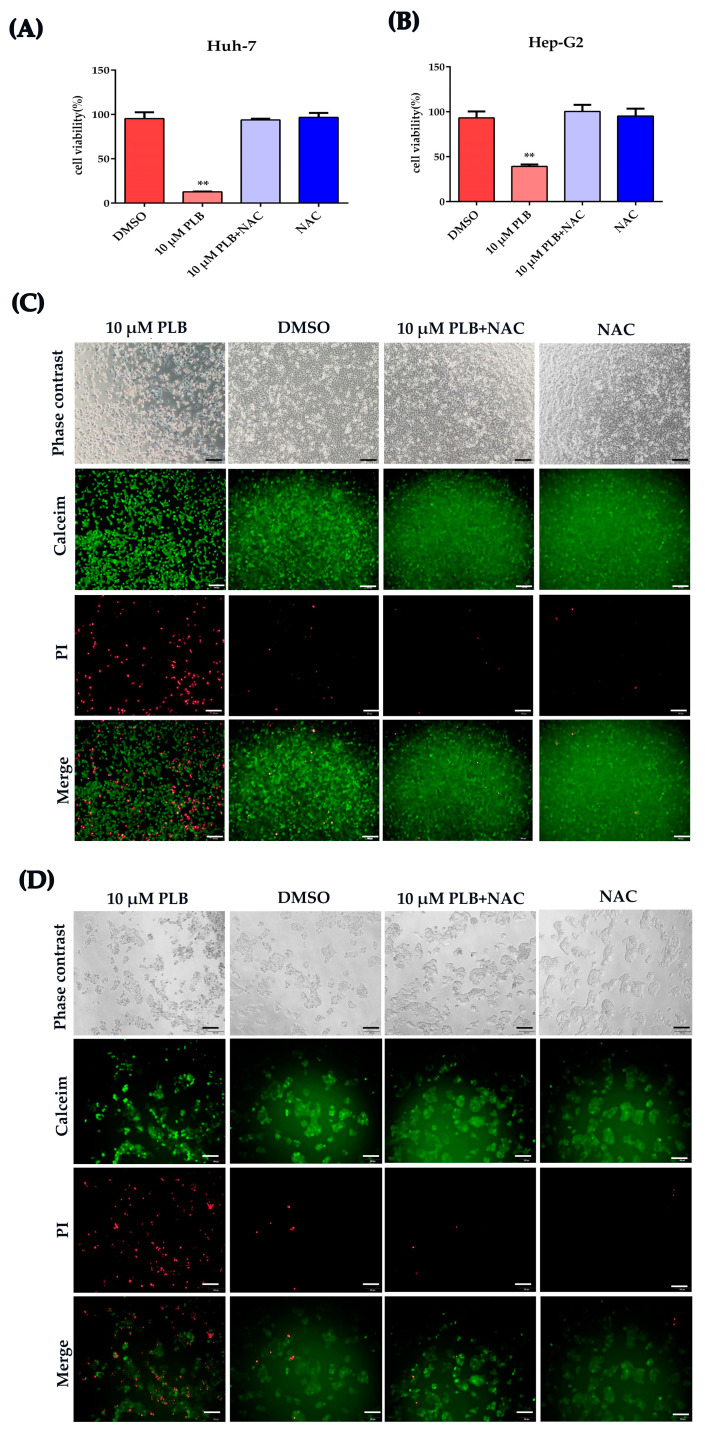
NAC impairs the cytotoxicity of plumbagin in HCC cells. (**A**,**B**) HCC cells Huh-7 and Hep-G2 were pretreated by 10 mM NAC for 1 h and subsequently treated by 10 μM PLB for 12 h. Cell viability was tested by CCK-8 kit (GlpBio, Montclair, CA, USA). Five replicates were set for this test and three independent experiments were performed. The data are shown as the mean ± SD, ** *p <* 0.01 compared to the DMSO group. (**C**,**D**) The live or dead cells were stained using the Calcein-AM/PI cell viability/cytotoxicity assay kit (Beyotime, Shanghai, China). The scale bar represents 100 μm. Calcein-AM staining showed green fluorescence in living cells, while Propidium Iodide (PI)-stained dead cells presented with red fluorescence.

As summarized in several reports, PLB can significantly elevate the ROS level in many different cancer cells [8,13], which indicates its important role in PLB’s anticancer effect. It is widely accepted that the anticancer effect of ROS chemotherapeutics is due to the induction of oxidative stress and ROS-mediated cell injury in cancer cells [28]. Therefore, we tested the cell viability after cells were pretreated by NAC for 1 h. As shown in Figure 6A,B, cell viability seemed unaffected by PLB treatment when pretreated with NAC. Calcein-AM/PI cell viability/cytotoxicity assay was further used to evaluate if the cytotoxicity of PLB was impaired by NAC pretreatment. As shown in Figure 6C,D, NAC pretreatment could almost eliminate PLB’s cytotoxicity, since cell viability was not affected by PLB treatment for 12 h.

### 2.7. NAC Treatment Reverses the Cell Cycle Arrest Effects Induced by Plumbagin

**Figure 7:**
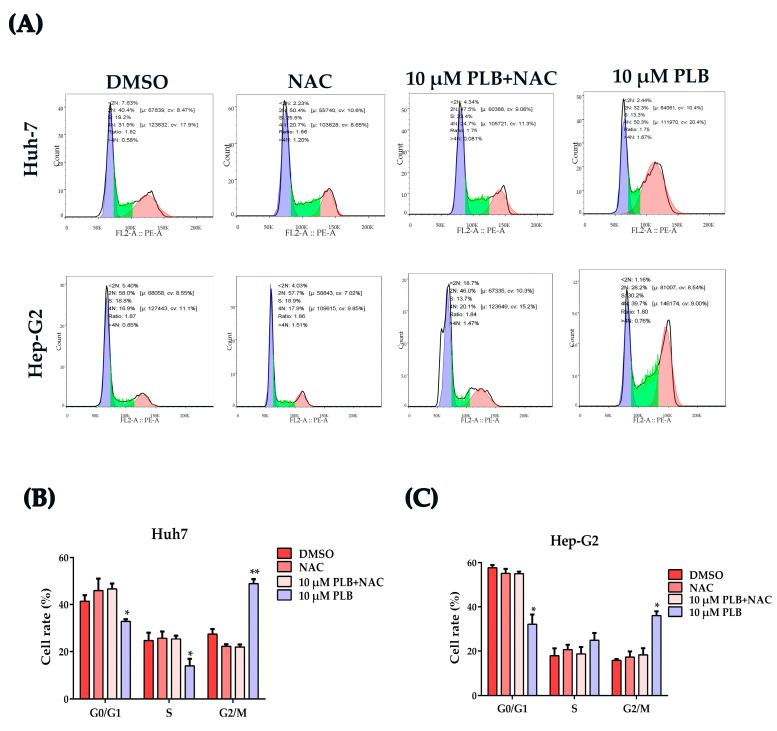
NAC pretreatment reverses the G2/M cell cycle arrest effects induced by plumbagin. (**A**) Representative images from the flow cytometry. Huh-7 and Hep-G2 cells were pretreated with 10 mM NAC for 1 h and were then treated with 10 μM plumbagin for 12 h, respectively. (**B**,**C**) Cell percentage at different cell cycle stages was analyzed based on the flow cytometry results; * *p <* 0.05, ** *p <* 0.01 compared to the DMSO group; three independent experiments were performed.

In order to evaluate the role of PLB-induced ROS regarding their cell cycle arrest effects, N-acetylcysteine (NAC), a potent ROS scavenger [29], was used to eliminate the excessive ROS induced by PLB, and cell cycle distribution was assayed as mentioned above. As shown in Figure 7, G2/M cell cycle arrest was not detected when cells were pretreated with NAC.

### 2.8. NAC Treatment Inhibits the Activation of ATM-Chk1/2-p53 Signaling Pathway

**Figure 8:**
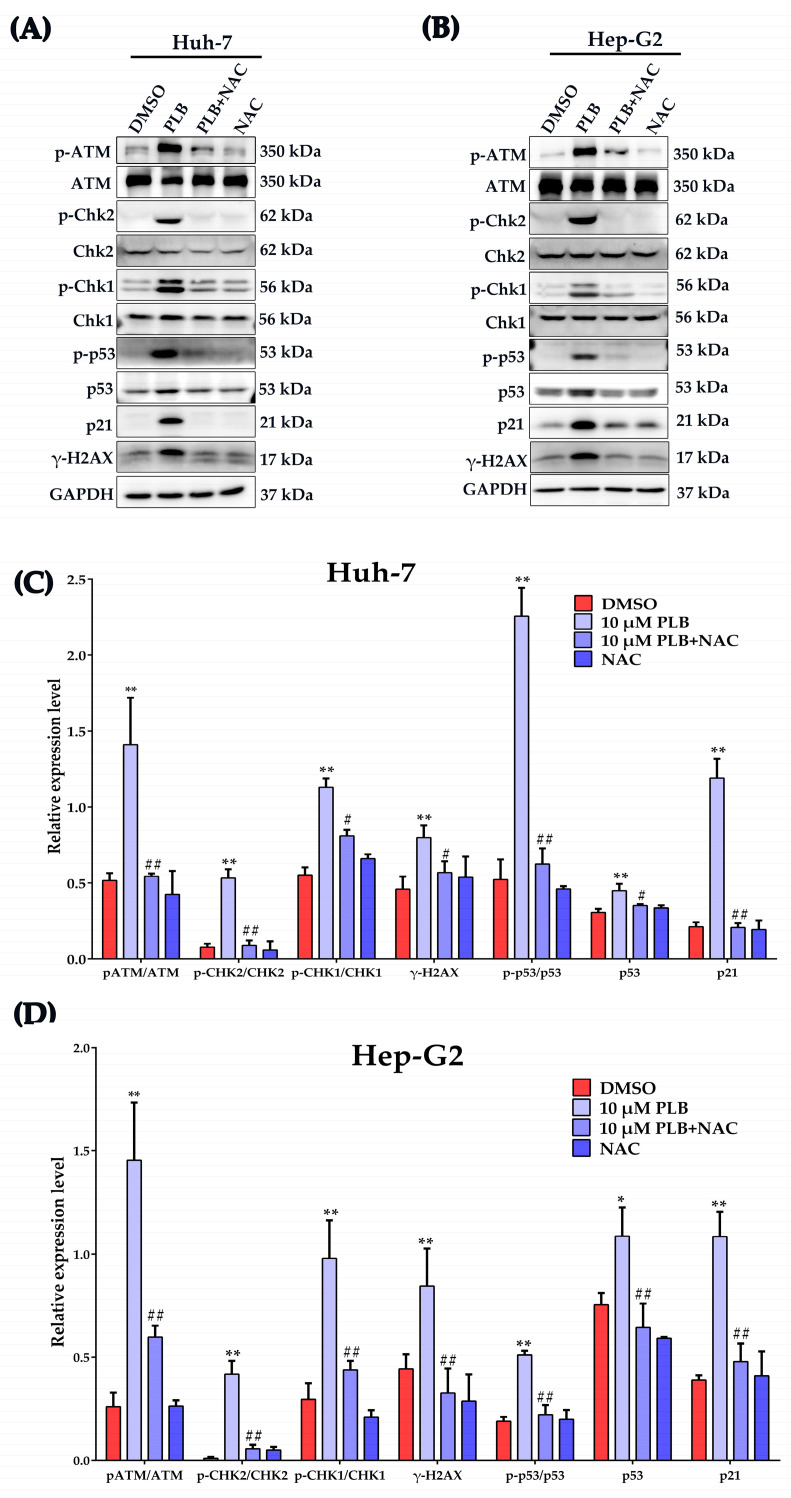
Activation of DNA damage response was inhibited by NAC pretreatment. HCC cells were pretreated with NAC (10 mM) or not for 1 h, and 10 μM of PLB was added into the medium for 12 h. (**A**,**B**) The expression of ATM, p-ATM (Ser1981), Chk2, p-Chk2 (Thr68), Chk1, p-Chk1 (Ser345), p53, p-p53 (Ser15) and p21 were determined by Western blot analysis. (**C**,**D**) Gray value statistical analysis of the indicated proteins. * *p <* 0.05, ** *p <* 0.01 compared to the DMSO group, # *p <* 0.05, ## *p <* 0.01 compared to the PLB treatment group; three independent experiments were performed.

In order to further investigate the involvement of ROS-mediated oxidative stress in the regulation of DNA damage response, HCC cells were pretreated by NAC (10 mM) for 1 h before PLB was added at the indicated concentration to induce DNA damage, as mentioned above. As shown in Figure 8A–D, NAC pretreatment could significantly inhibit the phosphorylation of H2AX by PLB. At the same time, the activation of DDR checkpoints such as ATM, Chk2 and Chk1 was also inhibited by NAC pretreatment. Meanwhile, the activation levels of the downstream cell cycle control checkpoint p53 and its downstream transcriptional factor p21 were shown to be significantly upregulated after PLB treatment, which could also be suppressed by NAC treatment.

### 2.9. PLB-Induced ROS-Dependent Downregulation of cdc25C

**Figure 9:**
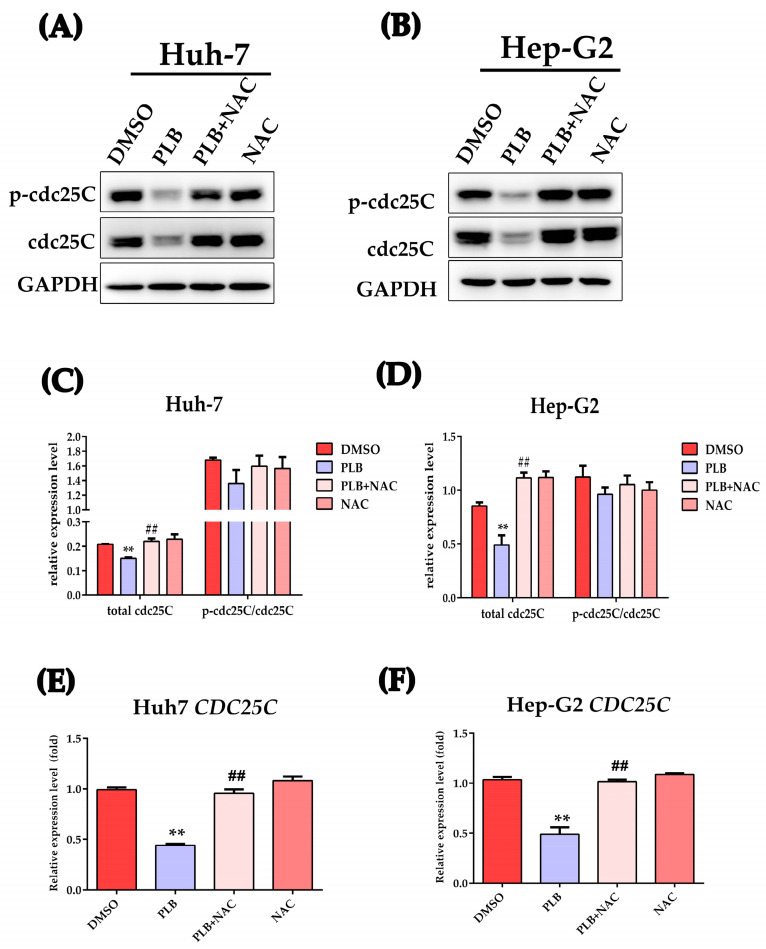
Plumbagin-induced ROS-dependent cdc25C downregulation. (**A**,**B**) HCC cells were pretreated with NAC (10 mM) or not for 1 h, and 10 μM of PLB was added into the medium for 12 h. The expression of cdc25C and p-cdc25C (Ser216) was determined by Western blot analysis. (**C**,**D**) Gray value statistical analysis of the indicated proteins. (**E**,**F**) Cells were treated as mentioned above and the relative mRNA levels of gene *CDC25C* were determined by the RT-qPCR method. ** *p <* 0.01 vs. compared to the DMSO group, ## *p <* 0.01 compared to the PLB group; three independent experiments were performed.

Activation of Chk1 and Chk2 could also inhibit cdc2 by inactivating cdc25C, the phosphate that normally activate cdc2 by removing the inhibitory phosphorylation, promoting the transition into M phase [19]. Western blot was used to test the effects of PLB on cdc25C level; as shown in Figure 9, the protein level was significantly downregulated after PLB treatment (Figure 9A–D). Meanwhile, NAC treatment could significantly restore the cdc25C protein level. In order to further investigate whether the reduced cdc25C was caused by transcriptional or post-transcriptional mechanisms, the qRT-PCR method was used to measure the *CDC25C* mRNA level. As depicted in Figure 9E,F, *CDC25C* mRNA transcription was significantly suppressed by PLB.

### 2.10. ATM-p53 Pathway Plays an Important Role in Plumbagin-Induced G2/M Cell Cycle Arrest

**Figure 10:**
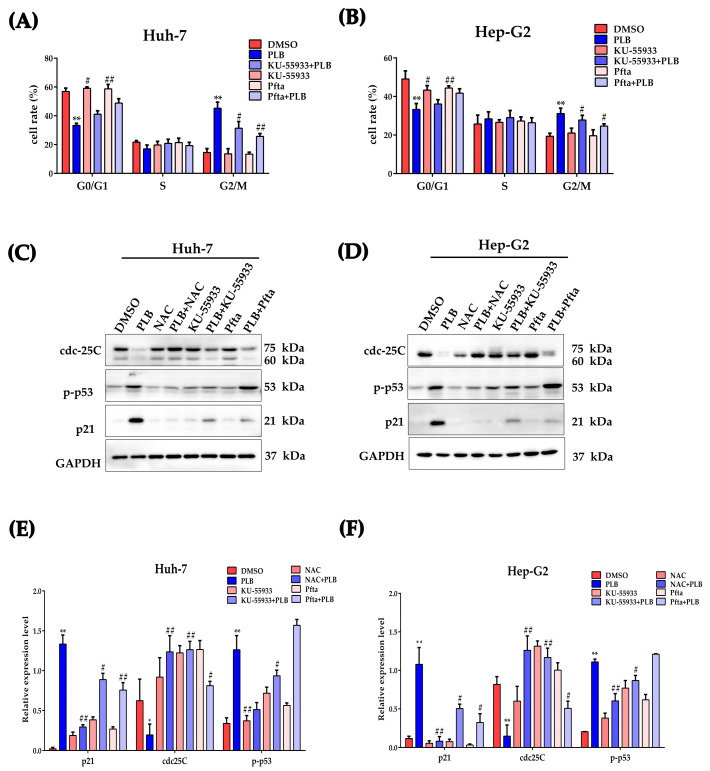
Involvement of ATM/p53 signaling pathway in PLB-induced G2/M cell cycle arrest. (**A**,**B**) HCC cells were pretreated with NAC (10 mM), KU-55933 (10 μM) and Pifithrin-α (Pftα,10 μM) or not for 1 h, and 10 μM of PLB was added into the medium for 12 h. Cell percentage at different cell cycle stages was analyzed based on the flow cytometry results. (**C**,**D**) The expression of cdc25C, p-p53 (Ser15) and p21 was determined by Western blot analysis. (**E**,**F**) Gray value statistical analysis of the indicated proteins. * *p <* 0.05, ** *p <* 0.01 compared to the DMSO group, # *p <* 0.05, ## *p <* 0.01 compared to the PLB treatment group; three independent experiments were performed.

ATM and p53 are generally considered key cell cycle regulators during DNA damage [30,31]. In order to further evaluate the role of ATM and p53 in PLB-induced G2/M cell cycle arrest, ATM and p53 inhibitors (KU-55933 and Pifithrin-α, Pftα) were used in this study. As shown in Figure 10, when ATM and p53 were inhibited, the cells arrested at G2/M significantly decreased compared to the PLB treatment group (Figure 10A,B). Western blot results showed that NAC, KU-55933 and Pifithrin-α could inhibit the downregulation of cdc25C when compared to the PLB treatment group. Moreover, the ATM inhibitor showed a more potent effect than the p53 inhibitor. Meanwhile, both ATM and p53 inhibitors could significantly suppress p21 expression (Figure 10C–F).

## 3. Discussion

Regarding the heterogeneity and complexity of disease progression, HCC treatment still faces major challenges. Thus, exploring safe and effective anticancer drugs and therapeutic regimens has become a major research topic. Phytochemicals obtained from plants play a very important role in both traditional and modern medicinal systems. In this context, bioactive components derived from traditional herbs may have the potential to overcome existing difficulties with HCC treatment, such as drug resistance, side effects during chemotherapy and even recurrence.

Previous investigations have shown that plumbagin exhibits a wide range of biological effects, including inhibiting proliferation and cytotoxicity, against multiple cancer cells, both in vitro and in vivo [8,13]. It is agreed that cancer cells are mainly compromised in their ability to exit the cell cycle rather than undergoing uncontrolled cell division [27]. Moreover, studies have also pointed out that most cell cycle control functions are also essential for cancer cell viability, and all cancers become increasingly dependent on remaining their cell cycle control mechanisms to prevent the excessive accumulation and propagation of genome instability. Intriguingly, inducing cancer cell cycle arrest seems to be a common mechanism or prerequisite of other effecting mechanisms employed by plumbagin [32,33,34,35]. Several studies have revealed that inducing G2/M cell cycle arrest can be an important mechanism for anticancer treatment [36]. In HCC cells, the ability to trigger G2/M cell cycle arrest with PLB treatment was observed in Hep-G2 and LM3 cell lines [22,24]. The mechanism by which this occurs has not yet been fully identified. In order to investigate the mechanism involved in PLB-triggered cell cycle arrest, the inhibitory and cytotoxicity effects of plumbagin on two HCC cell lines, Huh-7 and Hep-G2, were evaluated to determine the optimal concentration. As shown in Figure 1, plumbagin could significantly inhibit HCC cell proliferation and colony formation when the working concentration is higher than 10 μM. Cell cycle arrest was also observed in HCC cells when the PLB working concentration increased to 10 μM (Figure 2), which laid the experimental basis for the following mechanical investigation.

Cell cycle arrest usually occurs when cells encounter DNA damage. DNA in cells could be damaged by a variety of exogenous or endogenous insults, such as ROS, chemicals, radiation and free radicals, causing distinct forms of damage [37]. To some extent, the cancer cells’ fate depends on the degree of DNA damage. When mild DNA damage occurs, cells may repair the DNA lesions and recover using cellular DNA checkpoints and repair systems. However, if the damage is irreparable, cells can ultimately trigger cell death, such as apoptosis or necroptosis, to eliminate heavily damaged cells [20]. Thus, inducing excessive levels of DNA damage is more likely to result in catastrophic levels of genome instability and cell death in cancer cells than in healthy cells [38]. Plumbagin has been reported to be a potent inducer of ROS, an efficient DNA damage insult for cancer cells. As shown in Figure 3A, plumbagin treatment resulted in elevated levels of ROS in HCC cells. Furthermore, the level of cellular antioxidants such as glutathione rapidly decreased after PLB treatment, indicating excessive oxidative stress in HCC cells (Figure 3B–E). Recently, a computational analysis has shown that plumbagin may exert anti-HCC effects by enhancing ROS production and function as an upstream signal factor that regulates the PI3K/Akt/mTOR pathway, which is of critical importance in plumbagin-induced cell apoptosis and autophagy [16]. In lung cancer A549 cells, plumbagin could engender apoptosis via caspase-9 activation and ROS production [39]. In addition, ROS could also act as mediators or direct inducers of DNA damage. It is becoming increasingly widely accepted that the anticancer effects of many chemotherapeutics are due to the induction of oxidative stress and ROS-mediated cell injury, especially DNA damage in cancer [28]. In this study, IFA and Western blot results showed that plumbagin could induce severe DNA damage as the concentration increases (Figure 4). A similar observation was made in the study by Giovanna et al. (2020), using Hep-G2/C3A cells, where PLB was shown to trigger double-strand DNA damage (DSBs) [22]. In human breast cancer cells, plumbagin was also shown to induce DSBs in all the studied cell types, which may initiate compensatory and self-destructive mechanisms [40]. In this study, cells containing phosphorylated H2AX (γ-H2AX) foci clearly increased at high PLB concentrations. This result indicated that the main DNA damage pattern of cancer cells induced by plumbagin is DSBs, which may be a subsequent inducer of G2/M cell cycle arrest.

In case of DNA damage, cells had to evolve a toolbox of DNA damage response networks, including versatile sensing, signal transduction and execution systems, to handle the damage. The kinases ATM and ATR are DNA damage sensors at the apex of the cell cycle arrest signaling cascade. In response to double-stranded DNA breaks, cellular ATM was recruited to DSBs and autophosphorylates at serine 1981 (Ser 1981) and other sites to be fully activated [41]. After activation, ATM could phosphorylate its downstream transducers Chk2 and H2AX, which signal cell cycle arrest, apoptosis and DNA repair [42]. Chk1 is usually the target of ATR, which is often activated by single-stranded DNA damage. However, the substantial crosstalk between ATM-Chk2 and ATR-Chk1 makes Chk1 another target of ATM [19]. In this study, the Western blot results showed significant activation of ATM, Chk2, and Chk1, while the activation level of ATR was significantly decreased by PLB (Figure 5), indicating that ATM could be the activator of Chk1. A recent study found that breast cancer cells MCF7 and Hs578T were blocked at the G2/M phase by diosgenin-mediated Chk1 activation [43]. In another study, Timosaponin AIII was found to induce the breast cancer MDA-MA-231 and MCF7 cells arrested at G2/M phase due to ATM/Chk2 activation [44]. All these results suggest that Chk2 and Chk1 could both participate in G2/M cell cycle arrest. Regarding plumbagin, Chk2 activation has been reported in HCC and breast cancer cell lines [22,45]. The activation of Chk1 was first observed in this study. However, the detailed role that Chk1 activation plays during PLB-induced G2/M and its interplay with Chk2 requires more in-depth investigation.

ROS have been reported to play an important role in PLB-induced cytotoxicity, apoptosis and autophagy [16,23]. NAC was also found to protect against growth inhibition using plumbagin [46], indicating that ROS play a key role in PLB’s anticancer effects. However, the specific role of ROS in PLB-induced G2/M arrest has not been fully elucidated. As shown in Figure 6 and Figure 7, pretreatment with NAC could almost block the G2/M cell cycle arrest and cytotoxicity effects of PLB, suggesting that ROS could be the dominant triggers of PLB-induced G2/M cell cycle arrest.

The PLB-induced excessive ROS may lead to G2/M cell cycle arrest by inactivating the CyclinB-cdc2 complex via activation of the DDR pathway. Upon DNA damage, the key G2/M checkpoint p53 could be activated by ATM, ATR, Chk1 and Chk2, resulting in stabilization and accumulation [47]. Once activated, p53 could induce G2/M cell cycle arrest by inhibiting the activity of cdc2, the cyclin-dependent kinase required to enter mitosis. Under cellular stress or DNA damage, cdc2 activity could simultaneously be inhibited by three transcriptional targets of p53, p21, growth arrest and DNA damage-inducible-45 (GADD45), and 14-3-3 protein [31]. p21 represents the most important factor in p53-mediated cell cycle arrest [48], which can directly interact with and inhibit cyclinB/cdc2 complexes, resulting in cell cycle arrest. In this study, when pretreated with NAC, the activation of ATM, Chk1, Chk2, p53 and the DNA damage marker γ-H2AX was significantly inhibited (Figure 8). In addition, the expression level of the p53 target, p21, was also nearly blocked by NAC pretreatment. The activation of p53 may simultaneously participate in other cell death processes if the DNA damage level is irreparable [49]. In fact, the involvement of p53 in PLB anticancer mechanisms has been extensively reported. In breast cancer and osteosarcoma cells, PLB exerts anticancer activity through ROS-induced apoptosis via the p53-dependent pathway [50,51]. The previously reported network pharmacology result also indicated p53 as a top target of PLB in HCC [16,52].

Activated ATM/Chk2 or ATR/Chk1 could phosphorylate their downstream target, cdc25. Cdc25C is a phosphatase that removes the inhibitory phosphorylation of cdc2, which drives cell cycle progression into mitosis [53]. In this study, PLB significantly decreases the total cdc25C protein levels (Figure 9). However, we could not exclude the participation of the inactivation of cdc25C by Chk2 or Chk1. Similarly, NAC pretreatment could also block the downregulation of cdc25C, indicating that ROS also participates in this process. In fact, the levels or activity of the cdc25 family proteins has been found to be influenced by ROS. A similar report showed that Diallyl trisulfide induced G2/M arrest via ROS-mediated cdc25C destruction and hyperphosphorylation [54]. To elucidate the mechanism of cdc25C downregulation, the *CDC25C* mRNA levels were measured. As shown in Figure 9E,F, *CDC25C* mRNA levels were also inhibited by PLB. This is also observed in another ROS inducer, doxorubicin, which showed that DNA damage could stabilize p53 and suppress cdc25C and cdc2 expression [55]. The CDC25C gene contains a p53 binding site, and p53 could directly bind to the CDC25C promoter to induce G2/M arrest after DNA damage [56].There are two mechanisms by which p53 may inhibit CDC25C: first, the p53 response element inhibits the CDC25C promoter using the CDE/CHR element, and p21 also mediates DNA-damage-induced CDC25C gene down-regulation using this element. The second mechanism is related to the synergistic effect of Sp1-like cytokines and p53 [56]. In order to further evaluate the role of the ATM/p53 signaling pathway in PLB-induced G2/M cell cycle arrest, inhibitors (KU-55933 for ATM and Pifithrin-α for p53) were used to block the activation of ATM and transcriptional activity of p53, respectively. As shown in Figure 10, cell arrest in G2/M decreased after KU-55933 and Pifithrin-α pretreatment. The downregulation of cdc25C levels was also restored. These results further underline the importance of ATM and p53 in PLB-induced G2/M cell cycle arrest.

In summary, this study represents the first comprehensive investigation on the role of ROS-mediated DNA damage and the ATM-p53 signaling pathway involved in PLB induced cell cycle arrest, which underlines that ROS are the major weapon used by PLB to exhibit its anti-HCC effects and its potential clinical application. Although the anticancer effects and drug safety of PLB have been reported intensively, this compound has not been used in the market for cancer treatment. Poor water solubility and bioavailability represent major drawbacks for the introduction of PLB into clinical treatment. Moreover, a recent study reports that plumbagin damages testicular cells through the activation of the mitochondrial pathway involving the p53 protein network [57], which indicates its antifertility activity. Therefore, targeted drug delivery strategy is one of the optimistic ways for the clinical application of plumbagin, due to its target effects and limited toxicity. In fact, several breakthroughs have been made in recent years by loading plumbagin into nanoparticles or synthesizing biodegradable polymeric poly acid (PLGA) injectable gel implant of plumbagin, which was demonstrated to overcome the poor solubility, bioavailability and toxicity of plumbagin [58,59]. Future studies of PLB in such models would strengthen current findings and allow for clinical studies in HCC patients.

## 4. Materials and Methods

### 4.1. Cell Lines and Chemical Agent

Human hepatocellular cell lines Huh-7 and Hep-G2 were obtained from the National Collection of Authenticated Cell Cultures (Shanghai, China). Cells were maintained in Dulbecco’s Modified Eagle Medium (DMEM) (with 4.5 g/L D-glucose, 584 mg/L L-glutamine, 110 mg/L sodium pyruvate and 3.7 g/L sodium bicarbonate), and supplemented with 10% heat-inactivated fetal bovine serum (FBS) (GEMINI, Woodland, CA, USA) and 1% Pen Strep (Gibco, Waltham, MA, USA). The cells were cultured in an incubator (Thermo HERAcell 150i, Waltham, MA, USA) at 37 °C with a humidified atmosphere of 5% CO_2_. The PLB compound (2-Methyljuglone, 5-Hydroxy-2-methyl-1,4-naphthoquinone) was purchased from Sigma-Aldrich (CAS 484-42-5, MW = 188.18). PLB was dissolved in dimethyl sulfoxide (DMSO) as a stocking solution at 100 mM. ATM inhibitor KU-55933 (CAS No.:587871-26-9), p53 posttranscriptional activity inhibitor Pifithrin-α (CAS No.:60477-38-5) and ROS scavenger N-Acetylcysteine (NAC, CAS No.: 616-91-1) were purchased from Beyotime Biotechnology (Shanghai, China).

### 4.2. Cell Viability Assay

Huh-7 and Hep-G2 cells were plated in 96-well plates (2 × 10^3^/well) and treated with plumbagin at indicated concentrations for 12 h. Cell viability was measured using cck-8 kit (GlpBio, Montclair, CA, USA) following the manufacturer’s instructions, and the absorbance of each well was measured at 450 nm using a microplate reader (Thermo, Waltham, MA, USA). The half-maximal inhibitory concentration (IC_50_) was calculated.

Calcein-AM/PI staining kit (Beyotime, Shanghai, China) was used for cell viability/cytotoxicity detection. In brief, Huh-7 and Hep-G2 cells were plated on 24-well plates (3000 cells per well) and incubated overnight. Then, cells were treated by PLB at indicated concentrations for 12 h. After treatment, cells were washed by PBS and stained with Calcein-AM/PI detection buffer for 30 min. After staining, cells were washed twice with PBS buffer and observed under fluorescence microscope (Olympus, Tokyo, Japan).

### 4.3. Colony Formation Assay

A total of 1000 cells per well were seeded into 6-well plates with DMEM supplemented with 10% FBS and incubated in a humidified incubator at 37 °C with 5% CO_2_. The cells were treated with PLB at indicated concentrations and observed under a microscope every two days. At 7–10 days, the colonies were fixed in 4% paraformaldehyde, stained with 0.2% crystal violet and counted.

### 4.4. Cell Cycle Analysis

Cells (5 × 10^5^/well) were seeded in 6-well plates and treated with PLB at indicated conditions for 12 h. For cell cycle analysis, cells were digested by 0.25% trypsin and harvested by centrifugation. The harvested cells were fixed in 70% ice-cold ethanol overnight at 4 °C. After fixing, cells were washed twice with PBS buffer and labeled with propidium iodide (PI)/RNase Staining Buffer (BD, Franklin Lakes, NJ, USA) in the dark for 30 min and detected by flow cytometry (Beckman, Brea, CA, USA). The cell cycle distribution was analyzed by FlowJo^TM^ V10 software (BD Life Sciences).

### 4.5. DNA Damage Assay

The DNA damage of HCC cells was detected by a DNA Damage Assay Kit by γ-H2AX Immunofluorescence (Beyotime, Shanghai, China) according to the manufacture’s instruction. In brief, HCC cells were seeded into 96-well plates (3 × 10^3^/well) and treated with PLB at indicated concentrations. After 12 h treatment, cells were washed twice with PBS buffer and fixed with 4% paraformaldehyde. Then, the fixed cells were gently washed three times and blocked by QuickBlock^TM^ Blocking Buffer for Immunol Staining for 30 min at room temperature. After blocking, cells were incubated with rabbit anti-γH2AX monoclonal antibody overnight at 4 °C. For visualization, cells were further incubated in Alex Fluor 488-conjugated goat anti-rabbit secondary antibody (Beyotime, Shanghai, China) for 1 h and shielded from light. DAPI Staining Solution was used as a nuclear counterstain. A minimum of 50 cells in each experimental group were imaged using an inverted fluorescence microscope (Olympus, Tokyo, Japan).

### 4.6. ROS Assay

Reactive oxygen species (ROS) in HCC cells after PLB treatment were detected using the ROS Assay Kit-Photo-oxidation Resistant DCFH-DA kit (DOJINDO, Kyushu, Japan) according to manufacturer’s protocol. In brief, cells (2 × 10^3^/well) were seeded into 96-well plate. After overnight attachment, growth media was removed, and cells were washed twice with Hank’s Balanced Salt Solution (HBSS). They were then incubated with prepared working solution at 37 °C for 30 min, followed by treatment with 100 μL medium containing 0, 5 and 10 μM plumbagin for 12 h. The fluorescence intensity in each group was detected by an inverted fluorescence microscope (Olympus, Tokyo, Japan).

### 4.7. Measurement of GSH and GSSG

HCC cells were treated by PLB at indicated concentrations for 12 h. The total GSH and GSSG levels were determined by GSH and GSSG Assay Kit (Beyotime, Shanghai, China). All steps in this procedure were based on the instructions.

### 4.8. Western Blotting

HCC cells treated by PLB at indicated conditions were lysed by RIPA buffer on ice, and total protein contents were extracted. Protein concentration was determined by bicinchoninic acid (BCA) protein kit (GlpBio, Montclair, CA, USA). The samples were diluted with 5× SDS-PAGE Sample Loading Buffer and desaturated in boiling water for 10 min. Depending on the molecular weights of the target proteins, 6–15% SDS-PAGE gel electrophoresis was performed, and the separated proteins were transferred to PVDF membranes. Then, PVDF membranes were blocked with 5% skimmed milk for 1 h at room temperature. The PVDF membranes were incubated with the appropriated diluted specific primary antibodies overnight at 4 °C. The ATM, ATR, CHK1, CHK2 and p53 antibodies and the internal reference antibody anti-GAPDH monoclonal antibody were purchased from the Beyotime Biotechnology (Shanghai, China). The DNA damage response and cell-cycle-related antibodies were purchased from Cell Signaling Technology as a sampler kits (DNA Damage Antibody Sampler kit, Cat:9947 and cdc25C antibody Sampler Kit, Cat:9555). Theses membranes were further washed by TBST buffer three times and incubated with the corresponding HRP-conjugated secondary antibodies for 1 h at room temperature. Signals were visualized by enhanced chemiluminescence (ECL) (Millipore, Burlington, MA, USA). Image J software V1.8.0 (Rawak Software Inc., Stuttgart, Germany) was used to quantify the intensity of each band, and the values were normalized to the corresponding loading controls.

### 4.9. RT-qPCR Method

Total RNA in HCC cells treated by PLB at indicated conditions was extracted by NucleoZOL reagent (MACHEREY-NAGEL, Düren, Germany) and the 1st cDNAs were synthesized by GoScript™ Reverse Transcription Mix, Random Primers (Promega, Madison, USA). Specific primers targeting the *CDC25C* (Forward Primer: 5′-ACTTTGGCCTTCTGCTCAGG-3′; Reverse Primer: 5′-ACTTTGGCCTTCTGCTCAGG-3′) and *GAPDH* (Forward Primer: 5′-CAAATTCCATGGCACCGTCA-3′; Reverse Primer: 5′-GACTCCACGACGTACTCAGC-3′) gene were synthesized by Tsingke Biotech (Beijing, China). The relative mRNA levels of *CDC25C* gene were determined by qPCR method using GoTaq^®^ qPCR Master Mix (Promega, Madison, WI, USA).

### 4.10. Statistical Analysis

The experimental data are expressed as the mean ± standard deviation (SD). Statistical analysis was performed using SPSS 22.0. Paired samples *t*-tests were conducted for relative expression analysis. The graphical representations were performed using the GraphPad Prism 6 software (GraphPad Software Inc., San Diego, CA, USA). *p <* 0.05 was considered to indicate a significant difference.

## 5. Conclusions

**Figure 11:**
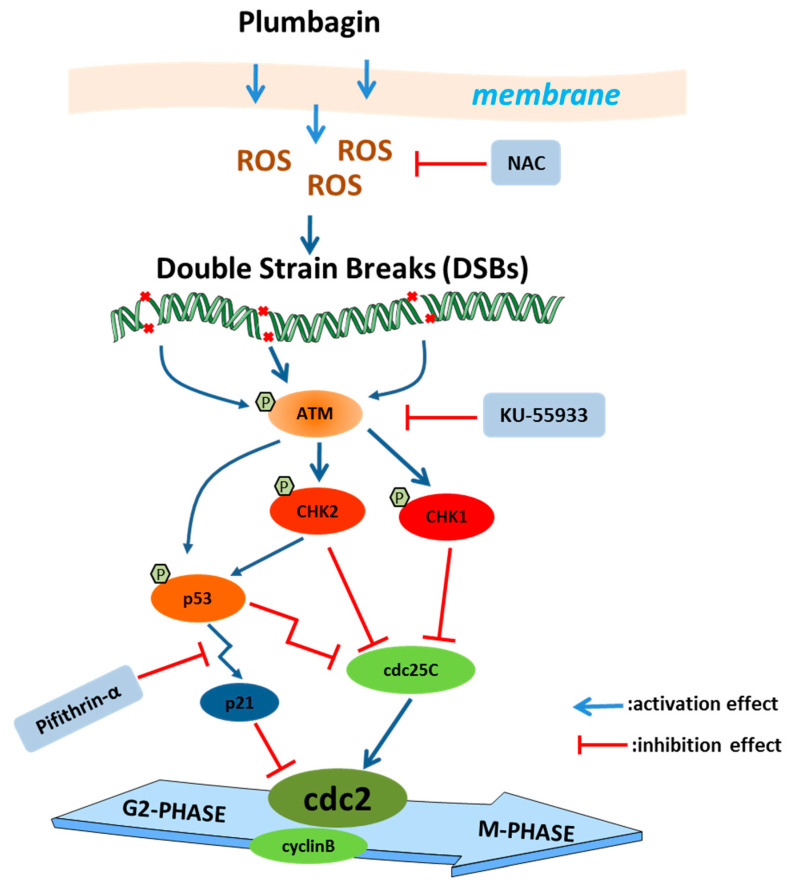
Presumed molecular mechanism of plumbagin-induced G2/M cell cycle arrest.

In conclusion, this research demonstrates that plumbagin can induce ROS-mediated oxidative stress, which acts as a trigger of DNA damage and ATM/p53 signaling activation. The activation of the DDR master kinase ATM transduces the signals further downstream kinase cascades involving Chk2 and Chk1. These kinases lead to the early inactivation of the target effector cdc25C by phosphorylation. However, p53 is activated and accumulated, which could serve as a transcriptional activator that promotes the expression of p21. The accumulated p53 could also suppress the transcription of the *CDC25C* gene. All these effects converge to suppress the activity of the cyclinB/cdc2 complex and cause G2/M cell cycle arrest (Figure 11).

## Data Availability

Not applicable.
